# General Medicine and Therapeutics

**Published:** 1895-08

**Authors:** 


### GENERAL MEDICINE AND THERAPEUTICS.

EDITED BY DR. LUTHER B. GRANDY.

### A PLEA FOR EFFICIENT LEGISLATION REGULATING MEDICAL PRACTICE.

[At the late meeting of the American Academy of Medicine, Baltimore, Dr. P. H. Millard, of Minnesota, read an able paper on this subject, and the Academy resolved that copies of it be furnished the leading journals, with request to publish. This paper is a masterly discussion of an important subject, and we take pleasure in inserting part of it in this place.—ED.]

During the last decade no question in medical sociology has attracted greater attention than medical education. The requirements of our colleges not being upon a par with those of other countries, nor with other departments of education in this country, it was but natural that the profession as a whole, the medical press, and organized bodies of medical men should join in a demand for needed reforms. During the formative period of our history it is but natural that abuses should have arisen in methods of education and obtain a firm rooting. A spirit of criticism exists that will not subside pending the definite determination of a question of such vital interest to the profession of the country.

As a nation during the first century of our history, we have established a system of common school education that challenges the admiration of the civilized world. It is a subject of regret, however, that in certain advanced lines of education our methods have proven most defective. This is true of medical education; a system having secured foothold with us that is indeed anomalous.

Having no support other than the fees of students; without university or college connection; without support from the State, generally accorded other systems of education; without restraining legislative enactments; without laws regulating the granting of charters for purposes of medical instruction; it is indeed little wonder that at the end of the first century of our history as a nation chaos should reign supreme.

The agitation of the question of medical education is bearing fruit, however, in that a majority of the schools situated in the Northern States demand at the present time evidence of preliminary fitness before matriculation, and that in a period of five years all colleges known to the writer have extended the period of time of study, with a change of the minimum length of term from five to six months. After the present year every medical school of recognized standing will require attendance upon four courses of lectures in different years, of six months' duration each course, before conferring the degree of M.D. The reforms thus far accomplished have only been secured in the face of determined opposition at the hands of the representatives of the low-grade institutions. Future opposition will result in disaster to the participants. Professional sentiment is decidedly with those schools now operating under the advanced curricula. This is particularly manifested by the increased number of matriculates in the last three years at schools operating under the four years' course. The fiscal matriculation at the University of Pennsylvania and Columbia is, approximately, eight hundred, Harvard five hundred, and others in proportion; while that of the recognized low-grade institutions have sensibly fallen off.

Notwithstanding the trend of public opinion, we are firmly of the conviction that our only safety consists in the establishment of efficient legislative acts in substantially every State. The high-grade schools are undergoing a period of evolution and are determined to inaugurate greater system in methods of work; with low-grade schools little evidence is at our command pointing to improvement.

The elevation of the standard of requirements in the latter class of schools has seemingly been entirely in response to the requirements of the respective State boards of medical examiners.

The indifference of the profession to methods of medical education has been far-reaching in its pernicious influences. Blinded by our own shortcomings, we did not awaken to a realization of our environment until our interests were greatly jeopardized. We found ourselves drifting, in the estimation of both the public and profession, towards a condition of professional inefficiency, not unlike that of French medicine in the seventeenth century, so graphically described by Molière. One of the greatest evils of our system was the flooding of our ranks with a horde of poorly educated practitioners far in excess of our legitimate demands. Following is a

TABLE INDICATING PROPORTION OF PHYSICIANS TO THE POPULATION.

| | |
|---|---|
| Austro-Hungarian Empire | 1 to 3,857 |
| Belgium | 1 to 2,841 |
| France | 1 to 2,666 |
| German Empire | 1 to 3,038 |
| Italy | 1 to 3,536 |
| Netherlands | 1 to 2,484 |
| Norway | 1 to 3,961 |
| Russia | 1 to 8,551 |
| Spain | 1 to 3,375 |
| United States | 1 to 500 |

The number of medical colleges indicates a similar disproportion.

NUMBER OF MEDICAL COLLEGES TO THE POPULATION.

| | |
|---|---|
| Austro-Hungarian Empire | 1 to 5,153,917 |
| Belgium | 1 to 1,534,111 |
| Brazil | 1 to 7,001,167 |
| Canada | 1 to 3,336,877 |
| Chili | 1 to 2,887,552 |
| France | 1 to 5,477,591 |
| German Empire | 1 to 2,471,923 |
| Great Britain | 1 to 2,358,767 |
| Italy | 1 to 1,455,109 |
| Netherlands | 1 to 660,249 |
| Norway | 1 to 1,988,771 |
| Sweden | 1 to 1,600,917 |
| Russia | 1 to 14,403,317 |
| Spain | 1 to 1,950,027 |
| United States | 1 to 440,151 |

It will be observed from the above that the proportion of practitioners and the number of schools are greatly in excess of other countries. Medical colleges in foreign countries are likewise independent financially, being, as a rule, directly supported by the State or possessing a direct university connection.

An investigation of this subject reveals beyond the possibility of successful controversion that the most efficient profession is found in those countries protected by efficient legislation; while a correspondingly low standard of professional fitness exists in countries not similarly protected.

At one time considerable opposition existed to the regulation of medical practice by legislative enactments. With the defeat of attempts to destroy the effects of this form of legislation by litigation and the moral support afforded by the recent decision of the Supreme Court of the United States and Supreme Courts of the several States, as well as the apparent benefits from the successful operations of the law in a large number of States, it is pleasing to note a decided change of sentiment in favor of this form of legislation.

The existing opposition to this form of legislation is rapidly disappearing, being greatly confined at present to the charlatan, the faculties of a few of our low-grade schools, and the public press. We can trace the existence of statutes regulating medical practice from the thirteenth century; in the year 1237 licenses were only obtainable in Italy upon attendance at medical lectures for a period of five years, with preliminary entrance requirements demanding three years' work in philosophy.

The first degrees in medicine were evidently conferred in Italy in 1384. Laws regulating medical practice have existed in all civilized countries for many centuries. It is unfortunate that in this country the diploma has been given a legal interpretation; in foreign countries it is simply an evidence of scientific value. With the advent of statutes regulating medical practice this custom upon the part of the courts is becoming abrogated. We cannot but conclude that in the older countries we have a superior profession in point of intelligence, with a more desirable environment; while with us we have, as a whole, men somewhat inferior in their preliminary training, a number triple that of any other country and a professional environment most undesirable.

The essentials of efficient medical legislation will incorporate the following features:

1. The adoption of more rigid rules governing the admission of students to medical schools.

2. The determination of the applicant's fitness to practice by an examination upon all the branches of medicine.

3. The right to refuse or revoke licenses for unprofessional or dishonorable conduct.

4. An adequate penalty for violation of the provisions of this variety of legislation.

5. The boards of examiners to be appointed by the governor, with proportionate representation by different schools of practice.

In support of demands for an adequate entrance requirement, it is conceded that medicine is now more nearly practiced from a scientific basis than at any time in its history. Without adequate preliminary fitness the broad field cannot be grasped nor its practice intrusted to persons without well trained minds.

Persons contemplating medicine as an avocation should give the scientific branches particular attention in preparation. A thorough course in the scientific department of our better equipped colleges or universities will permit of the successful accomplishment of the course now provided in the four years' curricula in a period of three years. I fully concur in the position taken by Professor Vaughan, however, in that the classical course does not prepare the student in a manner that he can safely abridge the work now required in the four years' curricula. The necessity of a thorough college training is more apparent now than at any previous time. While an immediate attempt, looking to the demand as above suggested, would probably meet with defeat, I am of the opinion, however, that by concert of action we can secure the adoption at this time of an elevation of the standard of fitness, requiring a college or university matriculation, or its equivalent, of all students wishing to commence the study of medicine. If the student cannot furnish a matriculation ticket from a recognized college or university, he or she should be required to undergo an examination that would admit to such course.

Under existing relations we cannot safely intrust this examination to the representatives of the teaching body. Except in a few of our high-grade schools, the entrance examination has been a farce as at present conducted. The factors leading to this condition are the same as outlined earlier in this paper. It is the result of college competition with an unnecessary multiplication, in recent years, of the number of teaching bodies. It is my judgment, based upon a somewhat varied and extended experience, that a majority of the schools in this country exist to serve the personal interests of the respective faculties rather than to serve the legitimate demands of the people. About twenty-five per cent. of our schools have a matriculation of less than sixty pupils.

The determination of the fitness of the student to commence the study of medicine should be placed in the hands of a body of men entirely disinterested. I know of no body better qualified to superintend the execution of this important trust than a state board of medical examiners. If not such a body, then a committee composed of members of a faculty of a college or university.

The minimum of entrance requirements should be uniform between the different States. Under the operations of the New York law regulating the examination of students commencing the study of medicine, much good is being accomplished. I desire to urge upon the profession the necessity of provisions in future acts looking to a rigid protection of the gateway to the study of medicine.

Having submitted satisfactory evidence of preliminary fitness, only such persons should be permitted to undergo the professional test as have received their courses of professional education at schools of medicine whose curricula of requirements are acceptable to the respective boards. A minimum of requirements, both as to time and teaching facilities, are as essential in measuring professional fitness as it is for similar purposes in universities, colleges, and our public school system. A school should not be recognized unless it is working under a minimum that will assure the graduation of a class of persons that can safely be intrusted with the care of the sick. In arriving at a conclusion upon this most important function, I desire to particularly impress upon the members of these boards the fact that medicine as at present understood and practiced is radically different from that of a few years ago. To comprehend requires years of study and a training in laboratory methods and surgical technique that can only be grasped when afforded by a person trained in methods of medical pedagogy. The clinical and laboratory facilities of many of our schools are shamefully inadequate. Several colleges known to the writer have operated for years with substantially no assets. It is the duty of each board to inquire fully into the facilities of each school represented by graduates who are applicants for degrees.

Having determined upon the fitness of the school to afford satisfactory courses of medical instruction, applicants holding degrees from such institutions should be admitted and a further test of fitness demanded by requiring an examination upon all the recognized branches of medicine. These examinations should be conducted by number, be scientific, and of sufficient severity to assure the public a thoroughly educated profession. Students from the respective schools of practice should undergo an examination upon the same questions, no necessity existing for questions not primary in character.

Licenses should not be refused or revoked for other than gross unprofessional or dishonorable conduct. In criminal cases it is not well to anticipate the processes of criminal law. The latter feature of our legislation has been instrumental in protecting the people from the professional charlatan in several States. Its provisions should be incorporated in all statutes regulating medical practice.

Owing to the difficulty in securing indictments and the consequent tardiness of legal processes, the penalty for violations of the provisions of this form of legislation should be by penalties imposed by a justice or a municipal judge; the latter method has given satisfaction as far as I am aware. Reasonable efficiency upon the part of the officers of these boards have been awarded by a full compliance with the provisions of this form of statute in all instances. The governor should have the appointing power, being responsible for the successful operations of the different State boards. Experience satisfies us that the so-called mixed boards are doing satisfactory work and operating in perfect harmony. Seemingly no excuse exists for the duplicate boards operating in a few States. At present, approximately thirty States possess legislation regulating medical practice. Seventeen States have a form of statute that fails to recognize the diploma as evidence of fitness to practice; consequently they may be classed with those States operating under efficient acts. In the latter class of States I particularly desire to call your attention to the results of work thus far accomplished. In a paper read before this learned body, at Detroit, Michigan, in 1892, I suggested the future influences of these boards as most important in shaping the future medical education in this country. I submit data at this time confirmatory of the position then taken, and reaffirm my former suggestion that future legislation will in a great measure determine and govern the work of the teaching bodies of the country.

I am deeply indebted to the officers of the various boards for courtesies extended and regret that space forbids reference to many suggestions and conclusions arrived at in the work of the different boards.

Data have been obtained from the following named States: Alabama, Minnesota, Maryland, North Dakota, North Carolina, New York, New Jersey, Virginia, and Washington.

The subjoined table indicates briefly the work of these boards:

| State. | Examined. | Licensed. | Rejected. | Per cent. |
|---|---|---|---|---|
| Alabama | 647 | 558 | 89 | 86.2 |
| Maryland | 150 | 105 | 25 | 80.6 |
| Minnesota | 641 | 499 | 142 | 77.8 |
| New York | 967 | 797 | 170 | 82.4 |
| New Jersey | 447 | 417 | 30 | 95.5 |
| North Carolina | 615 | 508 | 207 | 71. |
| North Dakota | 81 | 76 | 5 | 93.8 |
| Virginia | 835 | 613 | 222 | 73.4 |
| Washington | 207 | 167 | 40 | 80.6 |
| Totals | 4670 | 3740 | 930 | 82.2 |

It will be observed that of four thousand six hundred and seventy persons examined but eighty-two and two-tenths per cent. were successful in securing a license. The nine hundred and thirty unsuccessful applicants have, we doubt not, principally located in States not protected by this form of legislation.

I am pleased to direct your attention to the good work of the Minnesota board. The first act regulating medical practice in this State became operative in March, 1883. It was the form of legislation at present in force in Illinois. It was in operation five years, being supplanted by the present law. The present act requires an examination of all persons commencing the practice of medicine and as amended by the last legislature, the minimum of requirements is changed, demanding that all graduates of later date than 1898, furnish satisfactory evidence of having attended at least four courses of lectures in different years, of not less than six months' duration each.

We have in Minnesota a practical illustration of the position taken in my former paper: "that in medical legislation we have the only solution of the problem of higher medical education." Having drafted these bills and by force of circumstances been somewhat conspicuously aggressive in urging their enactment, I have, in consequence, witnessed their operations with some concern and interest. The result is all that the most sanguine could have anticipated. In a period of twelve years the proportion of physicians to the population in Minnesota has been reduced from one practitioner to every six hundred and fifty in 1883 to one to every one thousand in 1895. The State has been substantially rid of the traveling charlatan.

The work of the New York board is attracting considerable attention. Notwithstanding pronounced opposition and many embarrassments, the act is destined to strengthen the character of the profession in this State. The Secretary, Dr. J. R. Parsons, in his 1893 report, says: "That the new law proves a barrier to the ingress of the incompetent, has operated to raise the standard of preliminary education, and improve the methods of teaching and terms of study of the different schools of medicine."

As this form of legislation becomes more fully understood and appreciated by the better class of schools, it will be observed as one of the most certain and reliable avenues of placing before the profession of the country the character of work being done in all colleges whose alumni apply for a license. A school doing honest work has little to fear at the hands of these boards; upon the contrary, as suggested in my former paper, it will be found that the proportion of applicants able to pass successful examinations will be a certain index of the character of instruction afforded students in the respective schools.

While the proportion of applicants successful is only eighty-two per cent., it will be found that, from the schools heretofore operating under a high grade of requirements, thus far at least in the work of these boards, nearly all graduates are successful in obtaining a license upon examination. In substantiation of this conclusion I again submit data, using therein the same schools as in my former paper.

The following table indicates the proportion of students successful on examination from alumni of schools heretofore operating under the three years' curricula:

| Colleges. | Examined. | Licensed. | Rejected. | Per cent. |
|---|---|---|---|---|
| Harvard | 31 | 31 | 0 | 100.0 |
| Columbia | 123 | 118 | 5 | 95.2 |
| Univ. of Penna | 126 | 123 | 3 | 97.6 |
| Univ. of Michigan | 83 | 78 | 5 | 94.0 |
| Northwestern Univ | 26 | 22 | 4 | 84.6 |
| Univ. of Minnesota | 149 | 148 | 1 | 99.2 |
| Totals | 538 | 520 | 18 | 96.4 |

I cannot but conclude, gentlemen, that efficient medical legislation will operate to bring about the following results, as applied to the profession and public:

1. It will protect the people by affording a profession of greater intelligence.

2. It will suppress charlatanry.

3. It will reduce the number of persons practicing medicine to a number commensurate with the demands of the people.

4. It will reduce the number of medical colleges, at present far above legitimate demands.

5. It will raise the general standard of professional fitness, assuring us a professional prestige in the future, becoming the most important of the learned professions.

In conclusion, we appeal to the profession to renew their efforts in securing efficient medical legislation, believing its operations will result most beneficially to both the public and profession.

### THE MANAGEMENT OF SUMMER DIARRHEAS IN INFANTS.

On this subject Dr. H. B. Allyn, of Philadelphia, has a good paper in the July *University Medical Magazine:*

As the food is the source of infection in most of the cases, the first thing to be done in the management of summer diarrheas in children is to inquire what food the child has been taking. It will generally be found that milk in some form is the basis of the food. A nursing infant will be fed from the bottle with milk none too fresh when first delivered, and before the milk has been swallowed by the infant it is spoiled. Often the nursling goes to sleep over a bottle without emptying it; when it wakes, the remains of the milk, which meantime has become sour, are swallowed. Or the milk itself may be wholesome and sweet, but the bottle and nipple not properly cleansed between the feedings. Contamination is especially liable to occur when a long tube is used to connect the nipple with the bottle.

Whatever the apparent connection between the milk and the illness, my experience has led me in every case to withdraw the milk absolutely for a time. The infant is better without any nourishment for twenty-four or forty-eight hours. The next thing after withdrawing all food is to empty the stomach and bowels promptly. For this purpose nothing answers as well as the stomach tube. When one is not at hand, a large-sized soft rubber catheter, attached through an intermediate glass tube and rubber drainage tube to an ordinary small funnel, answers every purpose.

The thing to be remembered in cases presenting marked toxemia is that prompt action is as important as in arsenical poisoning. Immediate action, therefore, is better than even a short delay to secure apparatus. In one case I induced the infant to drink a quantity of warm water, and then induced emesis by thrusting my forefinger down the baby's throat. In this case the infant lay comatose and collapsed all night, and only gradually roused during the succeeding forty-eight hours, but finally recovered after a tedious attack of entero-colitis. Evidently in this case only part of the poison was got rid of by emesis, and yet sufficient to save life.

The bowels are best emptied by a copious rectal injection of warm water given by the fountain syringe. This is not done simply to empty the bowel of fecal matter—nature may have done that sufficiently—but to get rid of any toxic matter which may still linger in the bowel in spite of nature's efforts at elimination. When the stomach and bowel have been evacuated mechanically, castor oil or calomel should be given to empty the small intestines, which, of course, have been unaffected by lavage and the enema. Castor oil is preferable to calomel, except when the stomach is irritable.

Following the exhibition of the laxative a spice plaster or mustard plaster, or similar stimulating application, should be made over the liver with a view to stimulating it to destroy toxines already in the portal circulation. If the infant is much prostrated or in collapse, ten drops of whisky in hot water may be given every hour. If there should be high temperature with great restlessness, cold applications to the head may prove grateful, although they are not so well borne as in adults. A hot mustard foot-bath often quiets marked nervous irritability and allays fever by inducing perspiration. A hot general bath answers the same purpose, and is to be preferred if convulsions occur.

As already stated, the infant is better without any nourishment for twenty-four hours. Often parents are unwilling to obey the physician's directions in this particular. If there is reason to suspect such distrust, it is wiser to order a bland food than to subject the little patient to the risk of getting something unwholesome. Under such circumstances, I am in the habit of ordering egg-water or rice-water. The former is made by stirring the white of an egg in a pint of water that has been boiled or filtered, or both, and then cooled. This water can be seasoned with a little salt, and if there seem need for it in the condition of the baby, or if the infant be found to take it better, a teaspoonful or two of brandy may be added to it.

Rice-water is the water in which carefully washed rice has been boiled and then strained out through a cloth. Both rice-water and egg-water can be administered in a bottle in the usual way. The infant, as a rule, is not at first particular what it receives, so it is fluid and cool.

After an interval of abstinence for twenty-four hours, the infant will require some food, and the choice should fall upon one of several which are likely not to ferment. The rice-water and egg-water are to be preferred for at least a few days, if the infant is in good flesh and the passages from the bowels are very frequent. I often add to this diet some beef juice made in the usual way—that is, a juicy rump steak is broiled quickly over a hot fire, so that the outside is browned, but the inside remains juicy; this is then minced and its juice squeezed out by a lemon-squeezer. A teaspoonful may be given every three or four hours, either alone or with the addition of ten drops of whisky or brandy. An infant originally in good flesh can be nourished on this diet for a week or ten days, if the condition of the bowels require it.

If the infant needs more nourishment, it is often well to give one of the farinaceous foods in water, with the addition of beef-juice.

Often the great feebleness of the infant makes it necessary to feed it a few drops at a time from a teaspoon or with a medicine dropper. In all acute illnesses, especially of children, the parents need to be impressed with the fact that it not only does no good but may do actual harm to give the baby more food than it can retain and assimilate. The test of the proper amount of food to give must, therefore, be the retentive power of the stomach and the appearance of the feces. If the food is undigested, there will be evidence of it in the stools.

As soon as the toxemia passes away, in cases that do not immediately recover, the frequency of the stools is the most constant source of danger. At first they are fecal, then greenish and watery, and finally contain mucus, muco-pus and blood, or are made up wholly of mucus and blood. Their number varies from twenty to forty in twenty-four hours. The frequency of the movements shows the existence of increased peristalsis. This must now be checked by opium. But the administration of opium or astringents before the stomach and bowels have been emptied mechanically and by purgative, or before so many movements have occurred that nature has accomplished the elimination of poisons which art should have done sooner, is dangerous, and may result in locking up poisons in the intestines and favoring fatal coma. Often there is a movement as soon as anything enters the stomach. The amount of opium given must depend upon the requirements of each case. It is preferable to give it separately from any mixture which may be ordered, lest at any time it may be desirable to lessen the opiate or stop it, but to continue the rest of the mixture. Personally, I prefer the deodorized tincture of opium, and usually give it in quarter-drop doses every one, two or three hours as seems to be best. For the nausea and the inflammatory condition of the bowels bismuth seems to give the best results. The salicylate seems to be better than the older subnitrate and subcarbonate. It needs to be given in large doses, as Dr. L. Emmet Holt insists. A very good combination is:

℞. Bismuthi salicylat .............................................. clx gr.

Mist. cretæ, q. s. ad ............................................ f ℥ ii.

M. Sig.—Shake well. Teaspoonful every two hours.

To this may often be advantageously added a drop or two of spirit of camphor in each dose. Camphor is astringent and a stimulant, while it helps to allay the cutting pains in the belly so often present from the movement of gas in the inflamed bowels. Belladonna is also of service for this purpose.

The bismuth needs to be kept up continuously for days, until the stools become thicker and less frequent. By degrees these desirable changes usually occur, the blood soon disappears entirely, but the mucus is often present for a long time. One can tell before a napkin is opened whether it contains mucus or not by the peculiar, sickening odor.

If the infant is much emaciated, inunctions of olive oil are decidedly helpful, and at the same time they are usually grateful to the patient.

As soon as the patient's bowels begin to improve, a cautious return to milk should be attempted.

While cow's milk is the most frequent cause of the summer diarrheas of infants, it is, nevertheless, the best substitute for mother's milk. It is generally most acceptable when peptonized. In the first place, the peptonizing powder must be fresh; when fresh it is as white as wheat flour, but when stale it becomes yellow and lumpy. In the second place, the milk and cream must be fresh. This I find the hardest thing to obtain. With the majority of our poor families it is practically, impossible. Unless the milk is fresh, it is folly to waste money in peptonizing it; the milk will be sour before the process is complete, especially if the longer processes are used. In the third place, the directions for peptonizing must be carried out with care, and the milk must then be placed in clean bottles, corked, and kept in a cool place.

There can be no question that when an infant is taking sufficient peptonized milk it is receiving ample nourishment; and in spite of the good results which have unquestionably followed the use of the different commercial foods, I always feel better satisfied when an infant which requires artificial food is taking peptonized cow's milk. As to sterilized milk, the results are not so uniformly good. The important point must not be forgotten, that sterilization alone lessens the digestibility and the nutritive value of milk; whereas peptonizd milk is both partly digested and therefore more readily assimilated than plain milk, and by being heated to 160° F. or above, becomes sufficiently sterilized to be free from all dangerous bacteria. Of course, sterilization even does not destroy chemical poisons. Sterilized milk at this stage of convalescence therefore seems inadmissible.

Under the leadership of Dr. T. M. Rotch, of Boston, an effort is being made towards a scientific adaptation of cow's milk to the needs of nursing infants. A milk laboratory is established which gets its milk direct from a dairy controlled absolutely by the laboratory. The milk and cream are separated in the laboratory and recombined in any proportion desired by the physician.

The cream separator gives a cream having a constant value of 16 per cent. fat, 4 per cent. sugar, and 3.6 per cent. proteids. The skimmed milk contains 0.13 per cent. fat, 4.40 per cent. sugar, and 4 per cent. proteids. A 20-per-cent. solution of milk-sugar is freshly prepared every day, and is used to modify the proportion of sugar in the cow's milk in accordance with the prescription of the physician. Thus, an average breast milk containing 4 per cent. fat, 7 per cent. sugar, and 2 per cent. proteids, could be approximated as follows:

| | |
|---|---|
| Fat, cream (16 per cent.) | f ℥ xii. |
| Sugar (20-per-cent. solution) | f ℥ xiiss. |
| Proteids, skimmed milk | f ℥ ix. |
| Distilled water | f ℥ xii. |
| Lime-water | f ℥ iiss. |

For eight feedings of six ounces each.

Rotch has found that one-twentieth of lime-water by volume is sufficient to neutralize the acidity of ordinary cow's milk, and that three and three-eighths drachms of powdered milk-sugar to eight ounces of modified milk are required to give it the sweetness of breast-milk.

This scientific method of Rotch is a great step in advance, and when milk so prepared comes within the reach of those who need it most in our large cities the sale of patent foods will largely cease. I am satisfied from personal experience that the greatest fault with the milk supplied in Philadelphia is that it is stale when it reaches the consumer. I doubt if very many poor persons receive the milk within forty-eight hours of the time the cows are milked, and with the great majority the time is much longer. In all probability, if the milk could be supplied to the children within twelve or even twenty-four hours of milking time, there would be fewer cases of summer diarrhea and a great saving in expense for artificial foods.

When the physician feels that the surroundings of his patient make it next to impossible either to get or to keep fresh cow's milk, it is far better to rely entirely on some artificial food or on condensed milk. While they are not as good as the best cow's milk, they are far safer for infants in summer time than milk which is not fresh to start with, or which cannot be kept sweet and free from contamination, owing to carelessness or poverty of the parents.

---

### IODINE MIXTURE NOT PRODUCING IODISM.

The following mixture will not produce iodism, though ten to twenty grains of potass. iodid. are given at a dose:

| | |
|---|---|
| Potassium iodide | oz. ss to oz. j. |
| Iron and amm. citrate | dr. ij. |
| Tr. nux vomica | fl. dr. ij. |
| Water | fl. oz. jss. |
| Tr. cinchona compound | fl. oz. ij. |

Teaspoonful in water after meals.

—DR. HARDAWAY in *Medical Week*.

